# Long-Term Follow-Up of Pediatric CNS Tumor Survivors—A Selection of Relevant Long-Term Issues

**DOI:** 10.3390/children9040447

**Published:** 2022-03-22

**Authors:** Maria Otth, Johanna Wyss, Katrin Scheinemann

**Affiliations:** 1 Division of Oncology-Hematology, Department of Pediatrics, Kantonsspital Aarau AG, 5001 Aarau, Switzerland; johanna.wyss@ksa.ch (J.W.); katrin.scheinemann@ksa.ch (K.S.); 2 Department of Oncology, Hematology, Immunology, Stem Cell Transplantation and Somatic Gene Therapy, University Children’s Hospital Zurich—Eleonore Foundation, 8032 Zurich, Switzerland; 3 Division of Oncology and Hematology, University Children’s Hospital Basel (UKBB), 4056 Basel, Switzerland; 4 Department of Health Sciences and Medicine, University of Lucerne, 6002 Lucerne, Switzerland; 5 Department of Pediatrics, McMaster University Hamilton, Hamilton, ON L8S 4K1, Canada

**Keywords:** pediatric cancer, central nervous system, long-term follow-up, multimodal treatment

## Abstract

*Introduction:* Survivors of pediatric central nervous system (CNS) tumors are at high risk for late effects and long-term morbidity. The quality of survival became increasingly important, as advances in diagnostics, multimodal treatment strategies, and supportive care have led to significant increases in long-term survival. *Aim:* This review aims to provide a global overview of the potential late effects and long-term follow-up care of CNS tumor survivors, directed to trainees and practitioners with less targeted training in pediatric oncology. *Late effects in CNS tumor survivors:* A specific focus on CNS tumor survivors relies on cognitive and psychosocial late effects, as they may have an impact on education, professional career, independent living, and quality of life. Further important late effects in CNS tumor survivors include endocrine, metabolic, cardiovascular, and cerebrovascular diseases. *Conclusions:* Comprehensive long-term follow-up care is essential for pediatric CNS tumor survivors to improve their quality of survival and quality of life. An individualized approach, taking all potential late effects into account, and carried out by an interdisciplinary team, is recommended, and should continue into adulthood. Existing recommendations and guidelines on long-term follow-up care guide the multidisciplinary teams.

## 1. Introduction

Pediatric tumors of the central nervous system (CNS) represent the second most common malignancy in childhood following leukemia, and are the most common solid tumors [1]. Pediatric CNS tumors show the highest cancer-related mortality in children independent of age at diagnosis, and survivors are at high risk for long-term morbidity [1,2,3]. The biological behavior of pediatric CNS tumors is very different depending on the entity. Glioma is the most frequent tumor entity in the pediatric age group and represents 50–60% of all CNS tumors. Its biological spectrum ranges from low-grade gliomas (LGG, WHO Grade I), often behaving like a chronic disease, to high-grade gliomas (HGG, WHO Grade IV), with a rather dismal long-term prognosis in most cases [4]. Medulloblastoma and ependymoma represent the second and third most common tumor entities. The most recent WHO classification of CNS tumors from 2021 comprises these pediatric relevant overarching groups: (1) gliomas, glioneuronal and neuronal tumors; (2) choroid plexus tumors; (3) CNS embryonal tumors; (4) pineal region tumors; (5) germ cell tumors; and (6) tumors of the sellar region [5]. Each group is again divided into several entities with age-specific distributions. Medulloblastoma or atypical teratoid/rhabdoid tumors (ATRT), for example, are much more frequent in infants and young children. On the contrary, germ cell tumors or craniopharyngioma are mainly diagnosed in older children and adolescents [4].

Progress in diagnostic approaches, including neuroimaging and molecular tumor characterization, and more elaborated treatment strategies, including neurosurgery, radiotherapy, and targeted drugs, contributed to an increasing overall survival [6]. Progress in supportive care further contributed to this increase in overall survival. The five-year survival rate of children aged 0–14 years diagnosed with low- and high-grade CNS tumors in the United States increased from ∼55% in the 1970s to 82.5% in those diagnosed in 2004–2016 [1]. The respective five-year survival rate of European children diagnosed with low- and high-grade gliomas, within the same age range, in 2000–2007, and recorded in a cancer registry, was 70% [7]. This number has to be interpreted with caution, as not all European cancer registries collected information on low-grade tumors. 

Due to the increasing survival of pediatric CNS tumor patients, emphasis has increasingly been placed on late effects and long-term follow-up care. As a consequence, different national and international long-term follow-up care guidelines have been developed, such as the Children’s Oncology Group (COG) guidelines in the US, the Dutch Childhood Oncology Group (DCOG) in the Netherlands, and the “Therapy based long-term follow-up” guidelines in the UK [8,9,10]. The recommendations in these guidelines span survivors of all tumor entities and are not specifically for CNS tumor survivors. The COG guidelines are categorized by treatment exposure, covering each chemotherapeutic agent, different radiation fields, surgical procedures, and hematopoietic stem cell transplantation separately. The guidelines from the Netherlands and UK are categorized by organ systems at risk (e.g., fertility, hearing). Independent of these structural differences, each guideline answers the questions on who needs screening, which screening test should be used, in which frequency the tests should be performed, and what should be done in case of abnormal screening results. The recommendations for CNS tumor survivors can therefore be found in the sections on chemotherapeutic agents, radiotherapy and (neuro-)surgery. All these guidelines allow one to define organ systems at risk for late effects based on the treatment received. For example, survivors treated with platinum agents are at risk for ototoxicity, fertility issues, renal toxicity, and peripheral sensory neuropathy [8]. The screening tests and intervals recommended for ototoxicity in this example are annual medical history and audiometry, where the frequency is based on current age, and whether additional cranial radiotherapy was applied or not. As some recommendations differ between the different long-term follow-up care guidelines in various degrees, the International Guideline Harmonization Group (IGHG) was launched, aiming to harmonize the different national guidelines and to solve the discrepant fields with evidence from systematic literature searches or the consensus of experts in the respective fields [11]. As the field of late effects is constantly growing, the COG and IGHG recommendations are updated in regular intervals, based on new evidence. In addition, new IGHG recommendations are constantly developed and added on the homepage [11]. All these efforts underline that comprehensive long-term follow-up care is needed for pediatric CNS tumor survivors to increase the quality of survival.

## 2. Treatment Modalities and Related Long-Term Issues in Pediatric CNS Tumor Survivors

The treatment of pediatric CNS tumors is generally multimodal, consisting of different combinations of surgery, radiotherapy, and chemotherapy, mainly platinum agents, antimetabolites, plant alkaloids, and alkylating agents.

Surgery is often the first treatment modality used and an integral part of the treatment of all CNS tumors. The extent of surgery depends on tumor location, and its relation to vital structures, and can be classified into three groups: (1) performing a biopsy to establish the diagnosis and to identify possible therapeutic targets, (2) removing tumor to reduce pressure, eliminating a vital threat and improve the quality of life, even if the tumor is not completely resectable, and (3) gross total resection. The paradigm of neurosurgical interventions is to remove as much of the tumor as possible without increasing morbidity and mortality. Progress in preoperative imaging and surgical planning, as well as intraoperative management with advances in neuromonitoring, neuronavigation, and minimally invasive approaches by microscope and endoscope led to better tumor removal and less injury to adjacent structures [12,13]. The prognostic relevance of the extent of resection is higher in CNS tumor entities for which less postoperative treatment options are available, while more radio- or chemosensitive entities may be curable even with limited resections.

Radiotherapy, particularly in young children, is a known risk factor for neuropsychological impairments and secondary malignancies in the radiation field. Radiotherapy can either be applied by photon or proton beams. Proton radiotherapy (PRT) has a better dose conformality compared to photon therapy, spares healthy tissue, and leads to the potential advantage of fewer late effects [14]. However, PRT is not available in every country, and therefore not accessible for all children and adolescents diagnosed with CNS tumors. Child et al. compared neurocognitive and academic outcomes in long-term survivors treated with focal photon therapy (XRT) versus PRT or craniospinal irradiation (CSI) [15]. They could demonstrate that neurocognitive and academic long-term outcomes were less favorable in survivors treated with focal XRT compared to PRT. Survivors treated with CSI showed the largest impairment, regardless of the radiation modality used [15]. Kahalley et al. assessed neurocognitive outcomes in pediatric CNS tumor patients who received PRT either focal or as CSI, versus surgery alone. Patients treated with focal PRT or surgery alone had no decrease in neurocognitive performance over 6 years, in contrast to significant declines in the CSI group [16]. To omit neurocognitive late effects, radiotherapy can be replaced by high-dose chemotherapy in very young children, resulting in only modest reductions in survival [17]. Unfortunately, certain CNS tumor entities such as ependymoma and very high-risk medulloblastoma continue to require radiotherapy to achieve acceptable survival rates.

Chemotherapeutic agents used to treat pediatric CNS tumors mainly consist of platinum agents (e.g., carboplatin, cisplatin), antimetabolites (e.g., methotrexate), plant alkaloids (e.g., vincristine, vinblastine) and alkylating agents (e.g., cyclophosphamide). These drugs have been used for decades in the treatment of childhood cancer. Due to their rather unspecific way of action and interactions with metabolic processes in healthy cells, they cause substantial acute toxicities and late effects, explained further below.

Advances in molecular diagnostics have led to the discovery of new tumor subtypes, incorporated in the most recent WHO classification of tumors of the CNS, and resulted in profound prognostic and therapeutic consequences [5]. Methylation profiles, transcriptomics, and whole genome sequencing led, for example, to the further subgrouping of medulloblastoma into four major, and ependymoma into nine subgroups [18,19,20,21]. Most pediatric low-grade gliomas, as a further example, are defined by alterations in the mitogen activated protein kinase (MAPK) pathway [22]. The detection of these alterations has led to the development of targeted therapies, directed against these kinases, for example, MEK and BRAF inhibitors [23,24,25]. The efficacy and safety of these agents, for example, dabrafenib or trametinib, are currently being evaluated in clinical trials and may have the potential to replace conventional chemotherapy as a standard of care for pediatric low-grade gliomas [26,27]. While targeted therapy trials for pediatric CNS tumors have had considerable success, immunotherapy remains a challenge in a group of tumors with a lower mutational burden compared to adult tumors. Nevertheless, a wide spectrum of immunotherapy trials including immune checkpoint blockade, vaccine therapies, chimeric antigen receptor (CAR) T cells, and viral therapies are under investigation [28,29]. With these new treatment modalities, we currently face a lack of knowledge and uncertainty in terms of the persistence of tumor control and long-term toxicity due to missing long-term data.

All modalities used in the treatment of pediatric CNS tumors harbor the potential to cause acute toxicities and late effects. Acute toxicities are hardly avoidable and may include, to various degrees, nausea, hair loss, weight loss, and infections. They mostly completely resolve following the completion of treatment. Late effects are often more significant, emerging months to years following the completion of treatment, and can last for a lifetime [6]. Therefore, quality of survival is a very important factor, already during the acute treatment, but especially during long-term follow-up care. It is determined by the presence or absence of somatic and psychological illnesses, including endocrine, metabolic, hearing, visual, cardiovascular, or renal impairment, fertility issues, secondary malignancies or neurological deficits, as well as neurocognitive, behavioral, psychological and social sequelae (Table 1) [30,31].

## 3. Most Relevant, Challenging and Less Known Late Effects in CNS Tumor Survivors and Related Recommendations

The focus of most CNS tumor survivors regarding late effects is in the field of cognitive issues. However, the other organ systems at risk for late effects also have to be considered, since only the good functioning of all organ systems contributes to a good quality of life. To ensure this goal, institutional requirements and a multidisciplinary team are needed [55]. The multidisciplinary team should consist of neuropsychologists, endocrinologists, ophthalmologists, orthoptists, ear–nose–throat physicians, neurosurgeons, radiation oncology, neurologists specialized in neuro-rehabilitation, occupational therapists and speech therapists—the leadership role should be reserved for a pediatric neuro-oncologist. The disciplines needed for each survivor should be decided individually by the team. Further desirable institutional offers should include school or vocational counseling, social workers, or fertility specialists. When the survivors reach adulthood, a planned transition into adult care with a clear transition process is recommended [56].

### 3.1. Neurocognitive Assessment

One of the potentially most serious late effects in pediatric CNS tumors survivors is the negative impact on neurocognition. Some survivors suffer from significant global deficits with decreased intelligence quotients or impaired language skills, and others from minor neurocognitive deficits, such as impairment in processing speed, executive or memory function, or academic performance, only detected by specific testing [6,15,57,58,59,60,61]. Risk factors for poorer neurocognitive outcomes include younger age at diagnosis, highlighting the increased vulnerability of the less mature brain to the tumor or treatment-related injury, as well as tumor location, the WHO grade of the tumor, and radiation therapy [62,63]. Chieffo et al. showed, in children diagnosed with supratentorial hemispheric tumors, that those with low-grade tumors had more deficits in neurocognitive function at presentation than those with tumors of higher grades [63]. Due to the slow-growing nature, and initially rather unspecific symptoms of low-grade tumors, time to diagnosis is often longer, resulting in children being exposed to chronic hydrocephalus or compression of important cerebral structures for a longer time. A further risk factor for impaired neurocognition is the “Posterior Fossa Syndrome” (PFS). PFS can occur after the surgical removal of tumors located infratentorially, in the cerebellum or fourth ventricle. PFS is clinically characterized by postoperative mutism, ataxia, signs of paralysis, and arterial hypotension. In particular, impaired executive brain function can persist for years following surgery [64]. The exact pathomechanism of PFS is unknown. Studies suggest that PFS may result from bilateral damage to the cerebellar-thalamic-cerebral tracts. As neuronal pathways including the cerebellum are involved in higher-order functions, these damages may also contribute to neurocognitive long-term issues [65,66].

The COG Long-Term Follow-Up (LTFU) guidelines recommend the assessment for neurocognitive deficits in survivors treated with methotrexate, high-dose cytarabine, or cranial irradiation. The assessment includes an annual history of educational and/or vocational progress and referral to a neuropsychologist for formal neuropsychological testing at entry into long-term follow-up care, and additionally, when neurocognitive deficits or educational concerns are identified [8].

To prevent new neurocognitive deficits, or to improve existing ones, several neurocognitive interventions are being explored today, including pharmacological and neurocognitive approaches. Conklin et al. showed that childhood cancer survivors treated with methylphenidate over 12 months performed significantly better in attention and behavioral tests than survivors not treated with methylphenidate [67]. Ayoub et al. tested metformin in a double-blind, placebo-controlled crossover pilot trial in 24 pediatric brain tumor survivors. Even though the authors did not aim to assess the efficacy of metformin in this population, they could show that survivors treated with metformin in the first phase of the trial performed better than those first receiving the placebo [68].

As interventions, neurocognitive training programs aim to draw benefit from training-related neuroplasticity of the brain [69,70]. Such training programs can be carried out, either within a consultation with a therapist [71,72], as training sessions to be performed at home [69], or as computerized cognitive training [70]. All three approaches seem to be feasible and efficacious in childhood cancer survivors in improving attention and increasing academic achievement to various degrees. Importantly, all four studies included only childhood cancer survivors diagnosed with neurocognitive deficits. Whether the described interventions can prevent neurocognitive deficits in survivors at risk without preexisting neurocognitive deficits cannot be answered. In addition, it is currently not known how these training programs contribute to long-term improvement in school and academic performance and satisfaction in daily life.

### 3.2. Endocrinological and Metabolic Assessment

In particular, CNS tumor survivors treated with radiotherapy, higher doses of chemotherapy, or those with a tumor located in the region of the pituitary gland, are at risk of developing endocrine or metabolic late effects [48]. Endocrine late effects can include hypothalamic-pituitary impairment, adrenocorticotropic hormone (ACTH) deficiency, hyperprolactinemia, precocious or delayed puberty, hypogonadism, impaired fertility and/or sexual function, low bone mineral density, metabolic syndrome, or hypothalamic obesity. The prevalence of self-reported endocrine late effects was 43% among brain tumor survivors who participated in the Childhood Cancer Survivors Study [49]. This proportion was 50% in a cohort of 114 brain tumor survivors after reviewing their medical records, with 60% of them having more than one endocrine disorder [73].

Growth hormone (GH) deficiency is the first and most common hypothalamic–pituitary disorder [48]. This may be due to the relative sensitivity of GH-releasing hormone neurons in the hypothalamus to radiation and pressure by the tumor (e.g., craniopharyngioma or optic pathway glioma). Abnormalities of the pubertal axis, clinically apparent as precocious or delayed puberty, or gonadotropin deficiency are also common. Less frequent are deficiencies of thyroid stimulating hormone (TSH) and ACTH [48]. An association has been found between the total radiation dose to the hypothalamic–pituitary area, and the development of each pituitary hormone deficiency [50,51,74]. Even though most endocrine deficiencies were identified within the first six years following the diagnosis of a CNS tumor in one study, screening should continue annually beyond these six years [8,75].

Alkylating agents (e.g., cyclophosphamide, ifosfamide) can cause ovarian or testicular damage, resulting in ovarian hormone deficiencies, reduced ovarian reserve, testicular hormonal dysfunction, and impaired spermatogenesis. Therefore, it is recommended to advise the patients or their parents about the possibilities of fertility preservation before the start of treatment [35,36]. During long-term follow-up care, referral to fertility specialists should be performed when hormonal issues are detected, or survivors have difficulties having their own children [8].

Metabolic syndrome can have a huge impact on the quality of life, morbidity and mortality of affected CNS tumor survivors. Cranial radiotherapy, brain surgery but also tumor location in the hypothalamic region (e.g., craniopharyngioma), can cause overweight, obesity, or a full picture of metabolic syndrome including hyperinsulinemia and dyslipidemia [52]. Damage to the hypothalamic region influences the regulation of the central clock negatively, causing altered circadian rhythm and a predisposition to metabolic changes, among other aspects. This results in an increased risk for cardiovascular and atherosclerotic disease and emphasizes the importance of regular screening for, and counseling regarding, a healthy lifestyle, and the avoidance of additional risk factors such as smoking.

Clinical examinations of survivors of pediatric CNS tumors should especially focus on anthropometric measurements (height, weight, BMI), pubertal development (Tanner stage, testicular volume) until maturation, sexual function, menstrual history, and menopausal symptoms annually [8].

### 3.3. Sensorineuronal and Visual Assessment

Sensorineural and visual issues can be initial symptoms, leading to the cancer diagnosis, and can be caused by the tumor itself. They are rather common during the initial presentation but often improve or disappear by treatment initiation. However, some symptoms can persist following treatment completion or even become apparent as treatment-related late effects after years to decades [6].

Ototoxicity has been reported by approximately 50% of childhood cancer survivors following platinum-based treatment, head- or CNS-directed radiotherapy, or both [76,77]. It typically presents as high-frequency hearing impairment, and might be accompanied by tinnitus [78,79]. Brain areas susceptible to radiation include the temporal bone with the middle- and inner ear and the brain stem. Older radiotherapy techniques are more likely to cause ototoxicity than current techniques (PRT) because crucial aural structures are exposed to higher radiation doses or scattered radiation [77]. Hearing impairment in children has to be detected early due to the adverse effect on speech, language and social–emotional development, and finally, academic performance [76,77,80]. Screening by reviewing medical history and pure-tone audiometry is recommended. The frequency of screening depends on the age at treatment/exposure, and whether additional radiotherapy was applied and ranges from every 2 to every 5 years. In case of abnormal findings, the survivors should be referred to a specialist [8,37].

Different parts of the visual system can be affected by the tumors themselves or the treatment. Impaired visual acuity can be caused by compression or invasion of the tumor into the optic nerve, the optic chiasm, or the visual pathway. Additionally, cranial radiotherapy may cause optic neuropathy or cataract [41]. Sellar tumors can cause visual field defects, resulting in hemianopsia. The palsy of cranial nerves innervating eye muscles can cause double vision. Some manifestations can be transient, resolve completely or partially, or persist as late effects.

Chemotherapy-induced peripheral neuropathy (CIPN), manifesting as sensory and/or motor deficits of various degree, is a common side effect of platinum agents and vinca alkaloids [81]. Neuropathic symptoms often resolve following treatment completion, but in some cases, they persist for years. Neuropathic pain and discomfort can cause high disease burden [42]. A recently published systematic review showed evidence that sensorimotor training remains the most crucial component in the treatment of CIPN [81].

### 3.4. Psychosocial Assessment

Studies on childhood cancer survivors have consistently reported that the subgroup of CNS tumor survivors are at highest risk for mental and psychosocial distress [82,83,84,85,86]. Young adult CNS tumor survivors are at further risk for delayed psychosocial and psychosexual development [83,87,88,89]. The main causes for poor psychosexual functioning in female CNS tumor survivors are endocrine deficits, including premature ovarian failure [88]. CNS tumor survivors show lower rates of close friendships, dating, cohabitation, or marriage, and non-independent living, representing an important milestone for satisfaction in daily life [86].

During treatment phases, children and adolescents miss considerable amounts of education, resulting in a disadvantage compared to peers. This is strengthened by several studies reporting that CNS tumor survivors are at higher risk for poorer educational achievement, with more frequent support by special schooling and a higher proportion of CNS tumor survivors being unemployed compared to controls [32,90,91,92,93]. Depending on the study, the controls were either siblings, peers, or non-CNS cancer survivors.

Regarding risky health behaviors, CNS tumor survivors show similar or only slightly lower rates than siblings and peers, despite their risk for, or aggravation of, existing late effects.

From these data, it is recommended to assess the educational or vocational progress and psychosocial aspects (e.g., signs of depression, anxiety, post-traumatic distress, suicidal ideation, social withdrawal, sleeping problems, persistent fatigue) at every visit in the late effect clinic, and to provide early support if needed [8,32,33].

Several training programs targeting social skills have been developed and evaluated to support CNS tumor survivors to improve different skills of psychosocial functioning, such as difficulties in peer relation or making friendships, resolving conflicts, and dealing with mobbing or empathy [94,95]. These training programs are either carried out in small peer groups, on larger scales involving schools, or as online training programs [94,95,96].

### 3.5. Cerebrovascular and Cardiovascular Assessment

Cerebro- and cardiovascular diseases represent the third leading cause of death in pediatric CNS tumor survivors, following cancer recurrence and secondary malignancies [97].

Stroke is a well-documented issue in CNS tumor survivors treated with radiotherapy [98,99,100]. Campen et al. showed a 100-fold higher incidence of stroke or transient ischemic attack (TIA) compared to the healthy pediatric population with a median time from first radiation to first event (stroke or TIA) of 4.9 years, with a median follow-up of 6.3 years [47]. In a report from the Childhood Cancer Survivor Study, nearly 50% of all detected strokes in childhood cancer survivors occurred in CNS tumor survivors, who represented only 13% of the study population [101]. They further showed that the risk to experience a stroke is additionally increased by the presence of hypertension or diabetes mellitus [101]. As previously mentioned, CNS tumor survivors are at risk of developing hyperlipidemia, diabetes, obesity, and metabolic syndrome [52]. These atherosclerotic risk factors need to be monitored and treated aggressively, including measuring weight, height, and BMI annually, and further examinations in the case of obesity. Additional cerebral vasculopathies following cranial radiation are Moyamoya, cavernous malformations, and cerebral microhemorrhages, with the main risk being bleedings and cognitive deficits [45,46,102].

CNS tumor survivors being treated with CSI may be exposed to scattered radiation to the heart, putting them at risk for cardiovascular disease. Ionizing radiation is known to cause an increased risk for cardiomyopathy, congestive heart failure, myocardial infarction, arrhythmia, atherosclerotic heart disease, valvular disease, pericardial fibrosis, and pericarditis [54,103].

In view of these data, long-term screening for cerebro- and cardiovascular disease among CNS tumor survivors is needed. Of equal importance is the education of CNS tumor survivors about their increased risk and potential symptoms (e.g., strong headache, acute word finding disorders or neurological deficits, dyspnea, palpitations). They should be encouraged to avoid behaviors that are known to increase the risk of cerebro- and cardiovascular disease, namely smoking and excessive alcohol consumption, and to control dyslipidemia, obesity, and diabetes [54].

### 3.6. Screening for Secondary Malignancies

Secondary malignancies (SMN) represent the second most common cause of death among adult survivors of pediatric CNS tumors [97]. Important risk factors associated with the development of SMN are treatment with radiotherapy and chemotherapeutic regimens including alkylating agents and anthracyclines. Chemotherapy-induced SMN is mainly leukemia, whereas radiotherapy is associated with solid malignancies [8]. Secondary gliomas (often high-grade gliomas), meningiomas, sarcomas, and thyroid cancer are the most common secondary solid malignancies in childhood CNS tumor survivors [43,44]. Survivors should be aware of their increased risk, and should be encouraged for risk-reduced behaviors and prevention strategies, including sun protection following radiotherapy. Regular physical examination including skin examination is crucial. The role of rigorously screening by imaging for secondary CNS malignancies is still a matter of debate, but CNS imaging has to be considered in case of clinical symptoms [104]. Earlier detection of high-grade gliomas as SMN does not influence the poor prognosis and survival rates of these tumors, whereas later detection of meningiomas (with typically good prognosis) similarly may not compromise survival [104].

## 4. Conclusions

Our review underlines that pediatric CNS tumor survivors need lifelong risk-adapted long-term follow-up care. They have, among all childhood cancer survivors, the highest risk for profound late effects [6]. Despite the success in increasing the survival rates of children and adolescents diagnosed with CNS tumors, the concomitant risk for late effects with possible impact in several areas of life is concerning. The physical and psychological late effects often align with a reduction in quality of life [31]. As a result, researchers and pediatric neuro-oncologists constantly aim to reduce the risk of these late effects by introducing novel, targeted therapies, and decreasing toxicity by omitting or reducing doses of radiotherapy and conventional chemotherapy.

To further improve quality of survival, lifelong follow-up care of CNS tumor survivors should be implemented in clinical practice. A bio-psycho-social approach is recommended and should be reflected in an interdisciplinary approach. Currently, the IGHG is constantly developing organ-specific, updated, and evidence-based guidelines, providing guidance on who needs surveillance screening, which tests should be performed, and in which frequencies should be measured [11]. Each pediatric oncology center and physician caring for childhood cancer survivors should be aware of the IGHG or similar guidelines. National guidelines, as mentioned in the introduction, are those from the COG in the US, the DCOG in the Netherlands, and the “Therapy based long-term follow-up” guidelines in the UK [8,9,10]. PanCare, the Pan-European Network for Care of survivors after childhood and adolescent cancer, also provides a set of surveillance recommendations [105]. All these guidelines are risk-adapted, based on the treatment received (surgery, chemotherapy, and radiation therapy), and cover all physical and psychological aspects of follow-up care. In addition, advising survivors and their families individually about potential risk factors and healthy lifestyles is of the utmost importance and should also be implemented in every late effect consultation. For the future, further intervention and prevention studies focusing on late effects need to be implemented.

## Figures and Tables

**Table 1:** Most relevant potential late effects in children and adolescents diagnosed with a CNS tumor (not exhaustive), according to the Children’s Oncology Group Guidelines [8].

Exposure	Potential Long-Term Issues
pediatric CNS tumor	adverse psychosocial/ quality of life effects
	mental health disorder [32]
	fatigue, sleep problems [33]
	neurological deficits due to tumor location
chemotherapy and cranial radiotherapy	dental abnormalities [34]
alkylating agents (e.g., Cyclophosphamide, Ifosfamide)	testicular hormonal dysfunction, impaired spermatogenesis [35] ovarian hormone deficiencies, reduced ovarian reserve [36]
platinum agents (e.g., Carboplatin, Cisplatin)	ototoxicity [37] nephrotoxicity [38]
antimetabolites (e.g., Methotrexate)	reduced bone mineral density [39]
	neurocognitive long-term issues, clinical leukencephalopathy [30,40]
corticosteroids	reduced bone mineral density [39], osteonecrosis
	cataract [41]
plant alkaloids (Vincristine, Vinblastine)	peripheral sensory or motor neuropathy [42]
radiotherapy	secondary malignancy [43,44]
cranial radiotherapy	neurocognitive long-term issues, clinical leukencephalopathy [30,40]
	cerebrovascular complications [45,46,47]
	hormonal deficiency [48,49,50,51] overweight, obesity, metabolic syndrome [52]
	cataract, ocular toxicity [41]
	ototoxicity [37]
spinal radiotherapy	artery disease [53]
	cardiac toxicity [54]
	scoliosis/ kyphosis
brain surgery	neurocognitive deficits [30,40]
	hormonal deficiency [48,49,50,51] overweight, obesity, metabolic syndrome [52]
spinal surgery	scoliosis/ kyphosis

## Data Availability

Not applicable.
